# Enhancing emotion regulation: investigating the efficacy of transcutaneous electrical acupoint stimulation at PC6 in reducing fear of heights

**DOI:** 10.3389/fpsyg.2024.1371014

**Published:** 2024-04-03

**Authors:** Lin Cong, Xiao Yu, Meiqing Huang, Jicheng Sun, Hao Lv, Taihui Zhang, Weitao Dang, Chaolin Teng, Kaiwen Xiong, Jin Ma, Wendong Hu, Jianqi Wang, Shan Cheng

**Affiliations:** ^1^School of Aerospace Medicine, Air Force Medical University, Xi’an, China; ^2^School of Biomedical Engineering, Air Force Medical University, Xi’an, China; ^3^Center for Military Medicine Innovation, Air Force Medical University, Xi’an, China

**Keywords:** transcutaneous electrical acupoint stimulation, fear of heights, PC6, emotion regulation, heart rate variability, virtual height stimulation

## Abstract

This study investigated the impact of transcutaneous electrical acupoint stimulation (TEAS) at Neiguan acupoint (PC6) on the physiological and behavioral responses of participants exposed in virtual height. 40 participants were included in the study and were randomly assigned to either a control group or an intervention group. Participants had an immersive experience with a VR interactive platform that provided somatosensory interaction in height stimulation scenes. Psychological scores, behavioral and cognitive performance, and physiological responses were recorded and analyzed. The results indicated that the intervention group had significantly lower fear scores compared to the control group. Analysis of heart rate variability revealed that the intervention group exhibited improved heart rate variability, indicating enhanced cardiovascular function and emotion regulation. The behavioral and cognitive results demonstrated that the intervention group exhibited higher left eye openness, faster reaction times, and greater movement distance, suggesting enhanced attentional focus, cognitive processing, and reduced avoidance behaviors. These findings suggest that TEAS at PC6 can effectively reduce fear and improve the regulation of physiological and behavioral responses to negative emotional stimuli.

## 1 Introduction

Fear is an emotion closely linked to evolution as an adaptive response to environmental threats. When the fear responses are triggered excessively in non-fearful situations, they may develop fear-related disorders in individuals. Fear of heights is one of the most fundamental basic survival responses, often accompanied by physiological and behavioral changes (Liu et al., 2021). People may exhibit a mix of autonomic, behavioral, and cognitive self-preservation responses when they anticipate danger, including breathing faster, heart rate increases, freezing, and avoidance (Borkar and Fadok, 2021; Christianson, 2021). In some cases, irrational or excessive fear of heights can develop into acrophobia, which can significantly interfere with normal daily activities (Arroll et al., 2017). Acrophobia is estimated to affect around 3.1–6.4% of the general population (Lebeau et al., 2010). Additionally, visual height intolerance (vHI) is also a distressing susceptibility to height stimuli that affects 28% of the population, with about half of those who are susceptible reporting a negative impact on their quality of life (Huppert et al., 2013). From susceptibility to height intolerance to acrophobia, there exists a continuum of cognitive and physiological responses to height exposure (Brandt et al., 2015).

Many therapeutic techniques focus on reducing or eliminating of fear in both humans and animals. Pharmacological treatments, such as protein synthesis inhibitors, have been used in animal studies to alter learned fear, which may have long-term effects on the modulation of fear memories and defensive reactions (Nader et al., 2000; Mckenzie and Eichenbaum, 2011). Such interventions cannot be used safely in humans due to a number of serious side effects, including leukopenia, lipid abnormalities, sleepiness, and weight gain. Apart from pharmaceutical interventions, non-pharmacological strategies have the potential to facilitate to eliminate fear responses as well. Device-based methods, such as transcranial direct-current stimulation, transcranial magnetic stimulation, and transcutaneous vagus nerve stimulation, have been found to improve extinction and alleviate fear responses (Burger et al., 2016; Pennington and Fanselow, 2018; Clarke et al., 2020). However, it is important to note that the effectiveness of device-based techniques in reducing fear may vary. Some research has shown that the application of a-tDCS did not significantly reduce feelings of fear in patients or animals (Manteghi et al., 2017; Wout et al., 2017). These conflicting findings could be attributed to differences in brain targets and stimulation frequencies used in the studies.

Cognitive behavioral therapy (CBT) is a commonly used non-pharmacological strategy that aims to modify maladaptive emotional responses by changing an individual’s thoughts, behaviors, or both (Kaczkurkin and Foa, 2015). Cognitive and behavioral flexibility allow us to adapt to different situations, shifting strategies as needed to meet changing environmental demands and also play a central role in evidence-based practice approaches for the treatment of fear and anxiety disorders (Powers et al., 2017). For example, attentional bias toward threat stimuli was found to improve in a study involving a single-session CBT for panic disorder (Kappelmann et al., 2020). About half of the variance in symptom change were explained by early reductions in attentional bias toward threat, which were found to predict better symptomatic improvement at a 1-month follow-up (Reinecke et al., 2013). Exposure therapy is one of the widely used CBT treatment method that has been found to be highly effective for specific phobias. Meta-analytical studies have shown that exposure therapy is more effective compared to no treatment, placebo treatment, and non-exposure-based active therapy conditions (Wolitzky-Taylor et al., 2008). These intervention strategies provide promising approaches for managing and reducing fear and anxiety. However, previous studies have focused on behavioral and the neural correlates of cognitive emotion regulation, and have not focused on investigating emotion regulatory strategies that directly involve the body, despite their effectiveness in clinical populations (Minewiser, 2017; Church et al., 2022; Menevşe and Yayla, 2023). Aside from the cognitive, behavioral and motivational concomitants, emotions have long been recognized as full-body events. Emotional Freedom Techniques (EFT) is a psychophysiological intervention that includes cognitive and somatic elements and it adds the novel ingredient of acupressure. Instead of using needles, practitioners stimulate acupuncture points by tapping on them with their fingertips (Stapleton et al., 2023). Extensive research on Clinical EFT has demonstrated its effectiveness in reducing symptoms of post-traumatic stress disorder (Clond, 2016; Sebastian and Nelms, 2017; Feinstein, 2022). Moreover, body tapping is also well suited for self-application in non-clinical settings because of its simplicity, such as sports performance, public speaking and university exams (Baker and Siegel, 2010; Church, 2010; Rahmi, 2013). The inclusion of body-based techniques in research and practice can provide a more comprehensive understanding and approach to emotion regulation.

Previous studies have primarily focused on emotion regulatory strategies, neglecting the necessary intervention duration. It may be due to the fact that reductions in fear and avoidance are easy to observe and measure, but they occur slowly. In many instances, clinical or experimental, the cognitive shifts are slow to develop, changing over weeks rather than minutes (Rachman, 2015). Several studies have found that EFT can lead to significant reductions in anxiety, even in a single-session application (Chatwin et al., 2016; Jasubhai and Mukundan, 2018; Dincer and Inangil, 2021). The visualization of body tapping as a means of emotional regulation has been shown to effectively alter immediate neural and behavioral responses to emotional stimulation by single-session (Wittfoth et al., 2020). Similarly, tapping acupoints has been shown to increase amygdala activation and decrease hippocampus activation in individuals with flight phobia (Wittfoth et al., 2022). It is suggested that EFT can be capable of regulating fear in the daily lives of healthy individuals, even with minimal time investment.

However, tapping acupuncture points with fingertips has limitations that restrict people’s actions and is unsuitable for situations that require physical manipulation. Because manual stimulation of acupuncture points can produce endogenous opioids, increase the production of neurotransmitters and regulate cortisol (Menevşe and Yayla, 2023). Cortisol is the main stress hormone and modulates the autonomic nervous system, reduces heart rate, pain, and anxiety through these neurochemical changes (Lane, 2009; Napadow et al., 2009). Therefore, acupuncture may have the capacity to modulate emotional reactions. But the acupuncture is an invasive intervention technology, it has certain safety risks when used in some scenarios with poor sanitary conditions. One study showed that transcutaneous electrical acupoint stimulation (TEAS) can reduce post-operative stress response and improve heart rate variability, with effects no different from traditional acupuncture (Zhou et al., 2020). TEAS is an emerging therapeutic approach that combines the effects of transcutaneous electrical nerve stimulation (TENS) with acupuncture point stimulation (Szmit et al., 2023). Some studies have shown that acupuncture at PC6 can relieve and control palpitations, and its effect is closely related to autonomic nervous function (Liu et al., 2020; Ye et al., 2023). Based on these findings, we hypothesize that applying TEAS at Neiguan (PC6) can produce a similar emotion regulation effect. This approach utilizes a portable electrical stimulation device that can automatically stimulate acupoints (Air Force Medical University, Xi’an, China) to implement TEAS. This would enable a more convenient and comprehensive intervention, potentially applicable in multiple dangerous situations such as aerial work, driving or flying.

## 2 Materials and methods

### 2.1 Participants

Potential participants for the study were recruited via public advertisement at the university, and were screened for fear of heights using self-reported fear scores (Likert 10). Exclusion criteria for participation included vestibular and balance deficits, as well as other neurological or orthopedic disorders that affect postural control. Additionally, the subjects were required to have experience with virtual reality but not have been exposed to virtual heights previously. The study included 40 male participants (age: *M* = 23.7, SD = 3.81), who were recruited as volunteers with estimated scores between 6 and 9 (targeting a height-fearful but non-clinical population). One participant were examined but had to be excluded due to technical problems. Each participant had normal or corrected vision. Prior to participation, all individuals provided written informed consent and received 150 RMB as compensation at the end of the experiment. The study was approved by the Ethics Committee of the Air Force Medical University.

### 2.2 Virtual height stimulation

**Figure 1:**
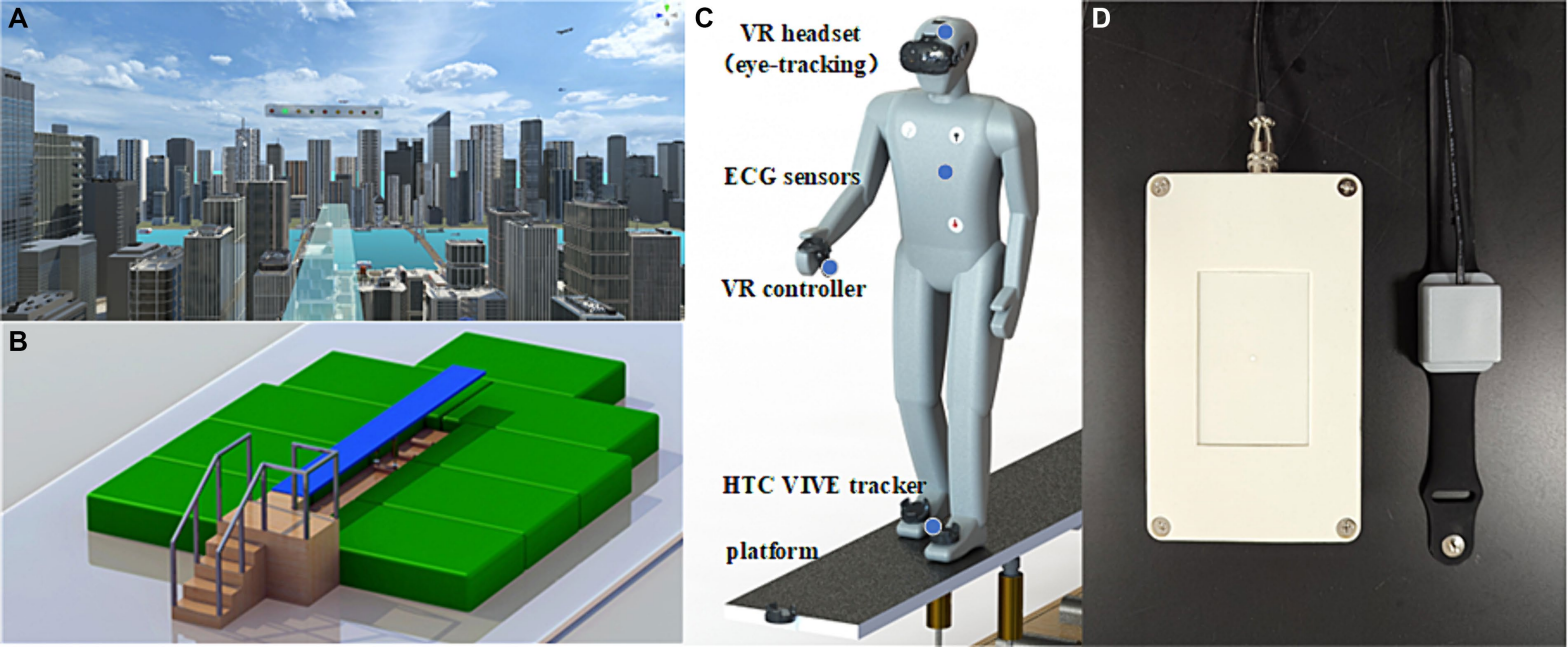
The schematic depiction of the experimental set up and the view of the diving board scene at 250 m above ground. **(A)** Participants were exposed to the virtual height scene using a HMD. **(B)** The VR interactive platform can provid somatosensory shaking through cylinder spring shock absorbers when participants walk on it. **(C)** Participants utilized VR controllers to complete cognitive tasks, and two HTC VIVE tracker were strapped to the instep of both feet to record movement distance. Additionally, three electrodes was used to monitor ECG by placing under the right collarbone, on the lower left costal arch, and on the lower left back. **(D)** The portable electrical stimulation device were used to stimulate PC6 during fear induction.

The virtual environment was created using the Unity3D engine and presented on an HTC Vive (HTC, Taiwan, China) with a 100° field of view, 1,080 × 1,200 pixels per eye, and a refresh rate of 90 Hz. Participants were exposed to the virtual scene via a head-mounted display and their head movements were tracked using an infrared positional tracking camera. Two HTC Vive base stations were used for 360° positional tracking, with the sensors positioned approximately 7 m apart from each other. The virtual scene depicted urban buildings and a continuous flow of vehicles (Figure 1A). After wearing the VR headset, participants stood on an elevated circular platform of the lookout tower. A transparent plate extended from the edge of the tower, allowing participants to observe the visual scene by moving their heads. The lookout platform is situated at a height of 250 m above the ground.

### 2.3 VR interactive platform

In order to realize the somatosensory interaction of VR in virtual height stimulation scenes, a carbon structural steel plate with a size of 4.8 m × 0.5 m is designed (Figure 1B). The plate has a modular design, allowing for quick and flexible deployment in various indoor and outdoor environments. The base is equipped with two cylinder spring shock absorbers, providing vertical freedom of movement for the plate. Since the two spring shock absorbers can work independently, they also provide the bridge floor with a certain degree of lateral freedom, allowing people to experience multi-angle stress shaking while walking. To facilitate VR device deployment, a special tracker installation slot is designed at the front end of the plate, enabling an immersive experience through synchronous combination of virtual and real elements. The corners around the plate are protected with flexible anti-collision cotton, and the ground is equipped with a safety cushion to ensure the safety of the subjects.

### 2.4 Psychological measures

Participants verbally reported their level of fear using a one-question 10-point Likert scale, ranging from 0 (no fear) to 9 (extreme fear). Additionally, the PANAS (Positive and Negative Affect Scale), consisting of 20 terms that describe different positive (e.g., active, inspired) and negative (e.g., scared, nervous) emotions, was also employed to evaluate fear and ensure the reliability of the scale score. The scale has reported internal reliability coefficients of alpha = 0.88 and 0.87 for trait positive affect (PA) and negative affect (NA), respectively, based on a sample of 663 university students (Watson et al., 1988). Participants rated each adjective on a scale from 1 (very slightly) to 5 (extremely) to indicate their feelings during the experiment. In this study, we calculated the sum of scores for the negative terms scared, nervous and afraid in order to evaluate the participants’ fear level.

### 2.5 Behavioral and cognitive measures

During the experiment, the participants’ position within the virtual environment (VE) was continuously tracked, and their movement distance (MD) was recorded using two HTC VIVE trackers that were strapped to the instep of both feet. The accuracy of the position data from the VIVE trackers has been found to be acceptable when compared to an optoelectronic 3D motion-capturing system, with an average deviation of less than 1 cm in position and an average rotation shift of approximately 1.6° (van der Veen et al., 2019).

Eye-tracking data was also collected using the HTC Vive Pro Eye with a built-in eye tracker, having an accuracy estimation of 0.5°-1.1° and a sampling frequency of 120 Hz. Some studies have used different hardware and reported information provided by the manufacturer to indicate the capability and usability of eye-tracking devices (Wroblewski et al., 2014; Ogura et al., 2019). In this research, the Vive SRanipal SDK is used to access non-filtered and filtered eye-tracking data. The embedded head mounted display (HMD) calibration system is used to calibrate the eye-tracking data for each participant. The raw data provided by the eye tracker includes the origin of the gaze, gaze vectors, eye openness, pupil diameter, and data validity. Our study used the saccade amplitude (calculated based on the origin of the gaze), open openness, and pupil diameter of both eyes for analysis.

To evaluate cognitive performance, a nine-light task which consisted of nine position points in a row with the colors of red, yellow, and green, was used to evaluate cognitive performance (Yang et al., 2023). The participants were required to press the corresponding button on the VR controller when a light was lit in every 5 s. The light would turn off when the correct button was pressed or after 3 s. The participants’ reaction time (RT) and accuracy rate (ACC) were automatically recorded during the experiment.

### 2.6 Physiological measures

Physiological indicators of fear or arousal during exposure to virtual heights were examined by monitoring participants’ electrocardiogram (ECG) using a Bluetooth physiological monitor (Tianjin Puray Instruments Ltd., Tianjin, China). The ECG was obtained using three Ag/AgCl electrodes placed under the right collarbone, on the lower left costal arch, and on the lower left back. To analyze heart rate variability (HRV), the ECG data was processed using Kubios software (v.4.1.0, HRV analysis, University of Eastern Finland). HRV indices were calculated, including 7 time domains, 4 frequency domains, and 8 non-linear parameters, as shown in Table 1.

**Table 1:** Summary of the parameters of HRV in this study.

Method	Parameter	Unit	Description
Time-domain	MeanHR	bpm	The mean heart rate
	SDNN	ms	Standard deviation of RR intervals
	RMSSD	ms	Square root of the mean squared differences between successive RR intervals
	NN50	beats	Number of successive RR interval pairs that differ more than 50 ms
	pNN50	%	NN50 divided by the total number of RR intervals
	HRVTi		The integral of the RR interval histogram divided by the height of the histogram
	TINN	ms	Baseline width of the RR interval histogram
Frequency-domain	lnVLF		Natural logarithm of absolute powers in the very-low-frequency band
	lnLF		Natural logarithm of absolute powers in the low-frequency band
	lnHF		Natural logarithm of absolute powers in the high-frequency band
	LF/HF		Ratio between LF and HF band powers
Non-linear	SD1	ms	In Poincaré plot, the standard deviation perpendicular to the line-of-identity
	SD2	ms	In Poincaré plot, the standard deviation along the line-of-identity
	SD1/SD2		Ratio between SD1 and SD2
	ApEn		Approximate entrop
	SampEn		Sample entropy
	DFA,α1		In detrended fluctuation analysis, short term fluctuation slope
	DFA,α2		In detrended fluctuation analysis, long term fluctuation slope
	D_2_		Correlation dimension

### 2.7 Experimental design and procedures

All participants were randomly divided into control group (20 subjects) and intervention group (20 subjects). We measured the physiological parameters of the two groups of subjects for 5 min prior to experiment, including heart rate, respiration, oxygen saturation, pulse, and skin temperature. The independent sample *t* test (data normally distributed) was used to analyze the physiological state of the two groups. There were no significant differences in any of the indices (*p* > 0.05), indicating that the physiological level of the two groups was consistent.

Before exposed in visual height, they were required to verbally report their level of fear, completed the PANAS and then equipped with VR headsets, physiological monitoring devices (Figure 1C), and a portable electrical stimulation (Figure 1D). Then, participants were given a message describing the concept of cognitive tasks in VR and practiced a 10-min nine-light task in a neutral virtual room to reduce the impact of task unfamiliarity. Following the practice session, participants were placed in the center of the VR tracking area and been exposed in the fear stimulation. They were asked to walk back and forth on the VR interactive platform with a fearful situation and complete the Nine-light task in 5 min. During fear induction, participants were given either intervention of TEAS or sham stimulation depending on their group. The control group also wore a portable electrical stimulation device during the experiment, but without receiving electrical stimulation. The stimulation methods used in this experiment were single pulse mode and continuous stimulation with a period of 5 s. After finishing the experiment, participants filled in the PANAS and verbally reported their state of fear again. Physiological, behavioral, and cognitive data were collected continuously during the experiment.

### 2.8 Data analysis

The normal distribution of the data was determined using the Shapiro–Wilk test and histogram. The independent sample *t* test were used for data sets with normal distribution, whereas the Mann–Whitney U test was used for data sets without normal distribution. Prior to conducting statistical analyses, the EEG signal were processed with a median filter and a 50 Hz notch filter to remove the baseline drift noise and power frequency noise, and then imported into Kubios HRV. The R-wave time instants are automatically detected by applying the built-in QRS detection algorithm. This in-house developed detection algorithm is based on the Pan–Tompkins algorithm (Pan and Tompkins, 1985). Kubios HRV detects artifacts with an automatic correction method which is more accurate and the method has been validated (Lipponen and Tarvainen, 2019). Artifacts are detected from a time series consisting of differences between successive RR intervals. During the analysis of HRV indices, two participants (one in the control group and another in the intervention group) had to be excluded due to loose electrodes resulting in poor ECG signal quality. Statistical analysis was performed using SPSS software, version 22.0, and a significance level of *p* < 0.05 was set.

## 3 Results

### 3.1 Subjective assessment results

**Figure 2:**
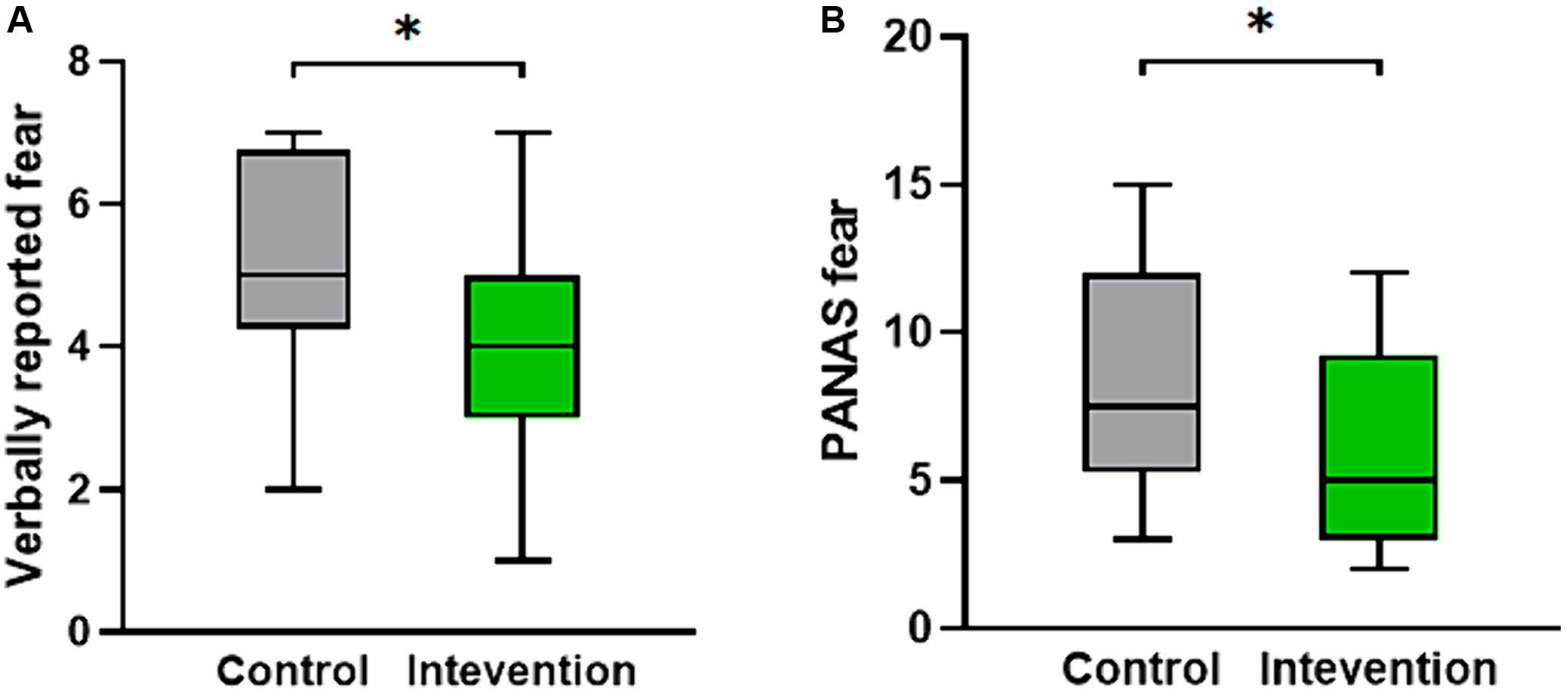
The distribution of psychological measures scores for both the intervention group and the control group. **(A)** The verbally reported fear score. **(B)** The PANAS fear score.*p < 0.05.

The scale data of the two groups after the experiment were statistically analyzed. The results indicated a significant difference in the verbally reported fear scores (*Z* = −2.309, *p* < 0.05). Figure 2A displays a box plot illustrating the verbally reported fear scores, showing that the intervention group had lower scores compared to the control group. Additionally, as depicted in Figure 2B, the PANAS fear score, which includes the negative terms score of scared, nervous, and afraid, also exhibited a significant difference, with the intervention group demonstrating significantly lower scores than the control group (*Z* = −2.265, *p* < 0.05).

### 3.2 Heart rate variability

**Figure 3:**
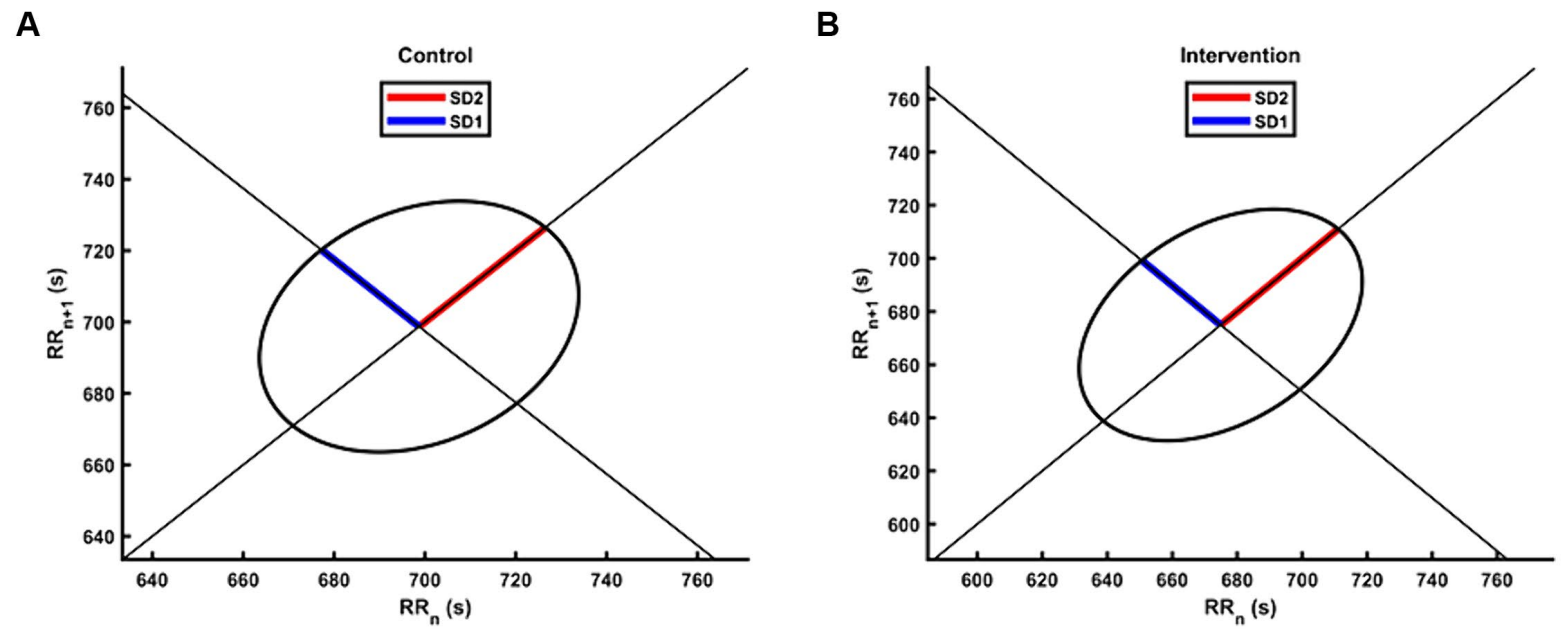
The Poincaré plot of HRV for the intervention group and the control group. The group-level scatters were not plotted due to the different number of RR intervals between participants during the 5 min experiment. **(A)** The Poincaré plot for the control group. **(B)** The Poincaré plot for the intervention group.

Table 2 presents the descriptive statistics and results of the participants’ 19 HRV indices in both the control and intervention groups. The analysis of time domain measures revealed that the intervention group had higher SDNN values compared to the control group (*Z* = −2.175, *p* < 0.05). Similarly, the TINN was also significantly different (*Z* = −2.584, *p* < 0.01), with the intervention group exhibiting higher values than the control group. In terms of frequency domain indices, the lnHF showed a similar pattern to SDNN, with significantly higher values observed in the intervention group (*Z* = −2.058, *p* < 0.05). In the non-linear measurements, the SD2 was significantly different (*Z* = −2.35, *p* < 0.05), with the intervention group demonstrating higher values than the control group. In contrast to SD2, the SD1/SD2 ratio in the intervention group was smaller than that in the control group (*Z* = −1.971, *p* < 0.05), resulting in a more distinct elliptical shape, as shown in Figure 3. There were no significant differences in other HRV indices (*p* > 0.05).

**Table 2:** Descriptive statistics and results of Mann–Whitney *U* tests of HRV indices in the control group and the intervention group.

Method	Parameter	Control Group (*N* = 19)					Intervention Group (*N* = 19)					*Z*
		M	SD	Q1	Med	Q3	M	SD	Q1	Med	Q3	
Time-domain	MeanHR	87.341	11.870	76.533	88.889	96.248	89.609	10.476	81.275	90.102	97.104	−0.598
	SDNN	35.306	9.700	28.247	31.593	38.842	43.727	14.603	33.667	42.046	50.092	−2.175*
	RMSSD	42.971	11.011	37.471	39.205	45.225	48.564	14.099	38.893	46.044	54.215	−1.445
	NN50	85.789	33.985	69.500	81.000	102.000	80.895	37.162	61.500	72.000	103.000	−0.701
	pNN50	21.576	9.785	15.502	18.790	26.554	24.380	10.014	17.568	20.189	29.914	−0.949
	HRVTi	8.969	1.950	7.699	8.820	9.644	9.582	2.260	7.681	9.091	10.846	−0.672
	TINN	185.895	50.329	151.000	165.000	223.000	243.263	80.588	192.500	226.000	272.500	−2.584**
Frequency-domain	lnVLF	4.267	0.789	3.576	4.437	4.820	4.611	0.585	4.177	4.438	5.033	−1.095
	lnLF	5.998	0.648	5.563	5.931	6.458	6.303	0.678	5.824	6.357	6.777	−1.387
	lnHF	5.527	0.703	5.027	5.592	6.070	6.058	0.819	5.459	5.964	6.606	−2.058*
	LF/HF	1.762	0.798	1.093	1.530	2.305	1.508	0.875	0.780	1.302	2.048	−1.182
Non-linear	SD1	30.424	7.799	26.529	27.752	32.027	34.402	9.988	27.548	32.599	38.388	−1.445
	SD2	39.288	12.216	30.333	34.777	44.592	51.115	18.993	36.973	50.227	56.040	−2.35*
	SD1/SD2	0.800	0.152	0.739	0.819	0.851	0.701	0.140	0.600	0.668	0.787	−1.971*
	ApEn	1.233	0.071	1.185	1.250	1.283	1.153	0.169	1.099	1.208	1.270	−1.299
	SampEn	2.010	0.193	1.920	2.057	2.146	1.898	0.291	1.786	1.919	2.112	−1.328
	DFA,α1	0.886	0.155	0.799	0.901	1.010	0.955	0.142	0.851	0.918	1.072	−1.182
	DFA,α2	0.421	0.132	0.347	0.421	0.478	0.407	0.114	0.336	0.389	0.463	−0.394
	D_2_	1.933	1.424	0.727	1.611	2.667	2.698	1.492	1.437	2.595	3.992	−1.679

1st quartile (Q1), Median (Med), 3rd quartile (Q3). The significance levels (two-tailed) are marked as follows: *p < 0.05, **p < 0.01.

### 3.3 Behavioral and cognitive parameters

**Figure 4:**
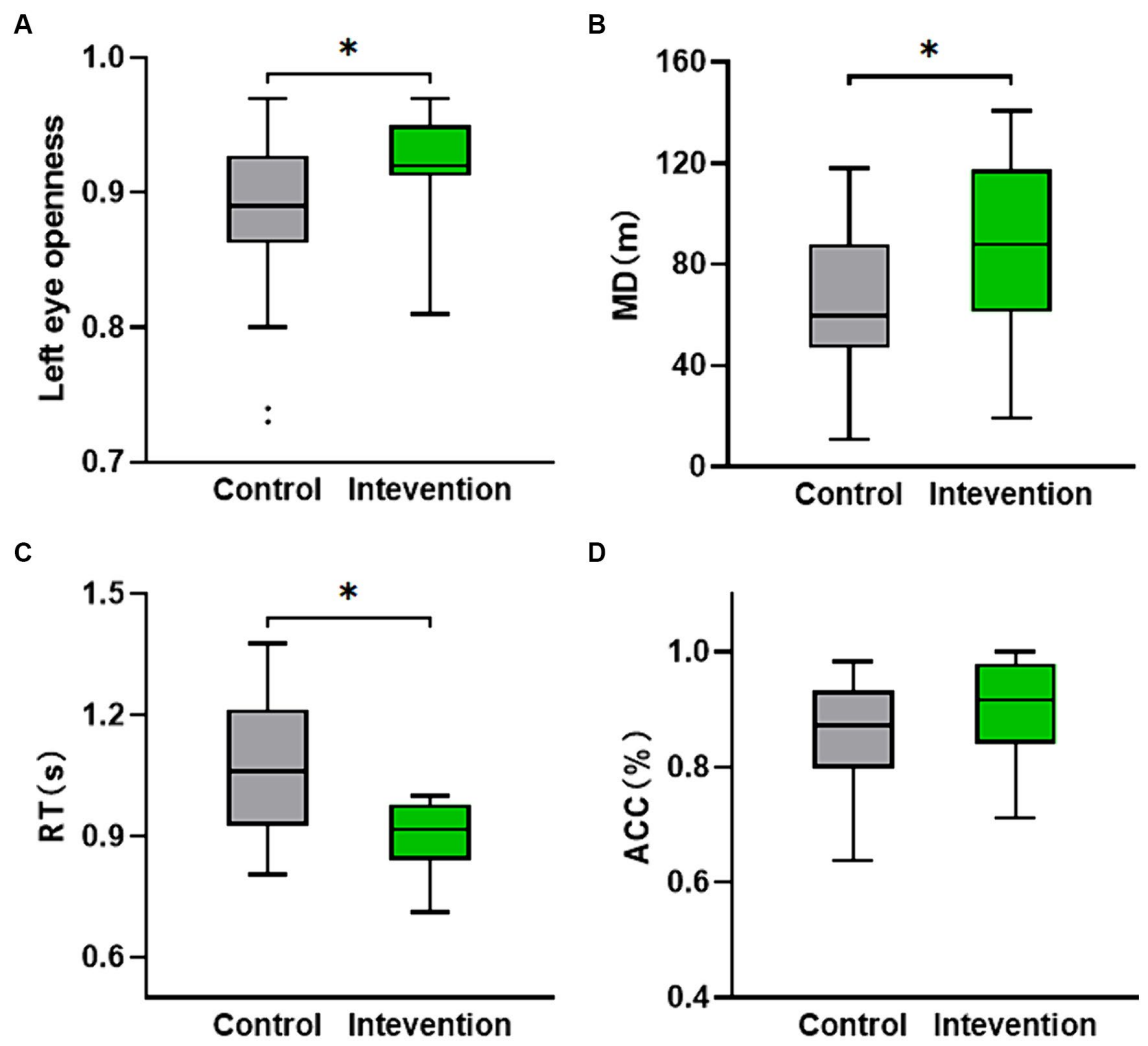
The distribution of behavioral and cognitive parameters values for both the intervention group and the control group. **(A)** Left eye openness. **(B)** Movement distance calculated from the participants’ location while walking on the VR interactive platform. **(C,D)** The nine-light task performance (ACC and RT). *p < 0.05.

Table 3 shows the eye movement parameters in both the control and intervention groups. The control group exhibited lower left eye openness compared to the intervention group (*Z* = −2.03, *p* < 0.05) as shown in Figure 4A, but no significant difference was observed in right eye openness. There were no significant differences in saccade amplitude and pupil diameter indices.

**Table 3:** Descriptive statistics and results of Mann–Whitney *U* tests of eye movement indices in the control group and the intervention group.

Parameter	Control Group (*N* = 20)					Intervention Group (*N* = 20)					*Z*
	M	SD	Q1	Med	Q3	M	SD	Q1	Med	Q3	
Left eye saccade amplitude	49.645	6.539	45.328	49.371	51.219	45.425	5.502	42.711	45.221	49.168	−1.731
Right eye saccade amplitude	46.114	4.563	43.277	45.559	47.583	47.022	4.873	43.941	45.973	48.791	−0.73
Left eye pupil diameter	4.331	1.020	3.581	4.357	4.778	3.840	0.496	3.558	3.699	4.145	−1.542
Right eye pupil diameter	4.211	1.162	3.166	4.343	4.941	4.211	0.775	3.625	4.255	4.746	−0.135
Left eye openness	0.882	0.064	0.864	0.889	0.924	0.919	0.042	0.914	0.917	0.951	−2.03*
Right eye openness	0.898	0.056	0.849	0.913	0.942	0.873	0.082	0.840	0.887	0.942	−0.649
Average of both eyes saccade amplitude	47.879	4.495	44.293	47.443	52.130	46.224	4.588	42.873	46.185	49.205	−1.19
Average of both eyes pupil diameter	4.271	1.079	3.303	4.390	4.868	4.026	0.575	3.593	4.020	4.590	−0.433
Average of both eyes eye openness	0.905	0.051	0.870	0.918	0.945	0.879	0.062	0.845	0.890	0.928	−1.313

1st quartile (Q1), Median (Med), 3rd quartile (Q3). The significance levels (two-tailed) are marked as follows: *p < 0.05, **p < 0.01.

The independent samples *t*-test was conducted to analyze the performance of the nine-light task (ACC and RT) and MD. Figure 4B displayed a significant difference in MD (*t* = −2.124, *p* < 0.05), with the intervention group (*M* = 84.783, SD = 36.171) demonstrating a higher value compared to the control group (*M* = 63.108, SD = 27.823). Figure 4C revealed a significant difference in RT (*t* = 2.323, *p* < 0.05), with the intervention group (*M* = 0.947, SD = 0.150) exhibiting a lower value than the control group (*M* = 1.064, SD = 0.167). Although not statistically significant (*p* > 0.05), the ACC of the intervention group was still higher than that of the control group as depicted in Figure 4D.

## 4 Discussion

In this study, we investigated whether TEAS at PC6 could effectively reduce fear exposed to the virtual height stimulation by single-session. The results demonstrated that the intervention had significant effects on reducing fear, as evidenced by changes in physiological responses and behavioral and cognitive parameters. These findings provide evidence supporting the use of TEAS as a means of emotion regulation in response to negative emotional scenes in healthy participants.

The significant differences observed in verbally reported fear scores and PANAS fear scores between the intervention group and the control group suggest that intervention was effective. These findings align with previous research highlighting the anxiolytic effects of acupuncture (Smith et al., 2018; Fu et al., 2022). It has been well-established that fear and anxiety often coexist and share common physiological and cognitive processes (Etkin and Wager, 2007; Mcteague et al., 2010). Anxiety is characterized by apprehension, worry, and anticipation of future threats, and it can intensify the fear response and prolong emotional arousal. Individuals with anxiety disorders exhibit heightened fear responses and are more prone to developing phobias (Lissek et al., 2009, 2010). Additionally, our findings are consistent with previous research on cognitive-behavioral interventions for specific phobias (Wang et al., 2022). The intervention of TEAS likely affected maladaptive thoughts and beliefs associated with the stimuli, resulting in a reduction in fear and anxiety.

HRV reflects the adaptability and flexibility of the autonomic nervous system and is particularly associated with cardiac vagal tone, which is relevant for various psychophysiological phenomena, including self-regulation mechanisms linked to cognitive, affective, social, and health (Thayer et al., 2009; Mccraty and Shaffer, 2015). Previous research has examined the association between HRV and emotion regulation strategies, suggesting that changes in HRV can reflect the effects of emotional interventions to some extent (Geisler et al., 2013; Zaccaro et al., 2018). In the present study, the control group exhibited lower SDNN values compared to the intervention group, indicating that the intervention had a positive impact on HRV, as reflected by higher SDNN values in the intervention group. Research has shown that SDNN is associated with various physiological and psychological processes. Higher SDNN values have been linked to better cardiovascular health, improved stress resilience, and enhanced emotion regulation (Thayer and Lane, 2009; Laborde et al., 2017). Conversely, lower SDNN values have been associated with increased risk for cardiovascular diseases, poor mental health, and worse anxiety (Quintana and Heathers, 2014; Larsson et al., 2023). Therefore, the intervention may indicate a potential improvement in cardiovascular function and stress regulation. Some researchers suggest using HRV indices that clearly reflect identified physiological systems with a theoretical underpinning, such as RMSSD, peak-valley, and HF (Laborde et al., 2017). The HF band reflects parasympathetic activity and lower HF power is correlated with stress, panic, anxiety, or worry (Shaffer and Ginsberg, 2017). In this study, the intervention group exhibited higher lnHF compared to the control group, indicating that TEAS may have an effect on the parasympathetic activity of the participants. As research suggests, higher vagal tone is associated with better executive cognitive performance and improved emotional and health regulation (Thayer and Lane, 2009; Thayer et al., 2012). Higher vagal tone has also been linked to better emotion regulation abilities, including the ability to downregulate negative emotions and upregulate positive emotions (Kok et al., 2013). These findings support our result that TEAS can increase vagal tone and enhance emotion regulation abilities. A wider distribution with greater variability will result in a larger TINN value, indicating a more irregular heart rate pattern. Conversely, a narrower distribution with less variability will yield a more regular heart rate pattern. The significant difference observed in TINN between the two groups may indicate that the intervention can lead to a more regular heart rate pattern.

Non-linear analyses may be more appropriate and accurate for HRV analysis, as the autonomic nervous system exhibits complex and irregular fluctuations (Piskorski, 2005). SD1 is supposed to be more sensitive to quick and high frequent changes whereas SD2 is viewed as an indicator of long-term changes. The ratio of SD1/SD2, which measures the unpredictability of the RR time series, is used to assess autonomic balance. In our study, the intervention group had significantly higher SD2 compared to the control group, while the SD1/SD2 ratio was significantly lower. According to our result, the vagus and sympathetic balance was affected by the intervention, improving cardiac autonomic modulation and reducing stress state. One study demonstrated that SD2 decreased significantly during a stress session compared to a control session, which was attributed to university examinations (Melillo et al., 2011). This finding supports the conclusion that higher SD2 may represent a lower state of stress.

Behavioral parameters, such as eye movement parameters and task performance, were also assessed in this study. Eye movements and spatial attention are closely interconnected systems that often shift together in many circumstances (Hodgson, 2019). The intervention group exhibited higher left eye openness compared to the control group, indicating a potential improvement in attentional focus and cognitive processing. One possible explanation for the increased left eye openness in the intervention group is that participants needed to intermittently look at the VR displaying buttons while using the VR controller with their right hand for task button interaction. As a result, the left eye observed toward the VR controller generated a greater offset, leading to higher eye openness. People direct their eyes toward objects of interest with the aim of acquiring visual information. However, processing this information is constrained by capacity, requiring task-driven and salience-driven attentional mechanisms to select a few among the many available objects (Souto and Kerzel, 2021). Thus, our observations suggest that attention, driven by TEAS, is biased toward cognitive tasks rather than fear stimuli. Although there were no significant differences in saccade amplitude and pupil diameter, the intervention group exhibited faster RT in the nine-light task and greater MD compared to the control group. The RT is influenced by the stages of cognitive processing involved in a task. Cognitive processing involves several stages, including perception, attention, memory encoding, decision-making, and response execution (Mittelstädt and Miller, 2020). Each stage requires a certain amount of time, and the cumulative time taken by these stages determines the RT. Emotion regulation has been found to have an impact on cognitive performance. Research suggests that individuals who are better able to regulate their emotions tend to exhibit improved cognitive functioning, such as enhanced attention, memory, and task performance (Gross, 2015). The smaller RT indicated that the intervention had a positive effect on reducing participants’ fear responses and improving cognitive performance. Furthermore, the greater movement distance observed in the intervention group suggests a reduction in avoidance behaviors and an improvement in coordination and execution of actions. Fear motivates different types of defensive behaviors and these defensive behaviors may in turn reduce, preserve, or amplify fear responding (Pittig et al., 2020). Based on the subjective scores and movement performance, we believe that participants in the intervention group experienced less fear, which led to lower avoidance behavior, manifested by an increase in walking distance.

## 5 Conclusion

Our study findings indicate that a single-session of TEAS at PC6 effectively reduces fear of heights. The results demonstrate that the intervention group had lower fear scores compared to the control group. Additionally, supporting evidence is derived from improvements in heart rate variability, increased left eye openness, faster reaction times, and greater movement distance observed in the intervention group. These findings suggest that TEAS has the potential to reduce fear, enhance cardiovascular function and emotion regulation, improve attentional focus and cognitive processing, and decrease avoidance behaviors. PC6 may be a suitable acupoint for regulating emotions in response to negative stimuli when using TEAS. However, further research is needed to fully understand the neural mechanisms underlying these effects.

## Data availability statement

The raw data supporting the conclusions of this article will be made available by the authors, without undue reservation.

## Ethics statement

The studies involving humans were approved by the Ethics Committee of the Air Force Medical University. The studies were conducted in accordance with the local legislation and institutional requirements. The participants provided their written informed consent to participate in this study.

## Author contributions

LC: Writing – review & editing, Writing – original draft, Visualization, Validation, Software, Project administration, Methodology, Investigation, Formal analysis, Data curation, Conceptualization. XY: Writing – review & editing, Writing – original draft, Methodology. MH: Writing – review & editing, Writing – original draft, Data curation. JS: Writing – review & editing, Writing – original draft, Methodology. HL: Writing – review & editing, Writing – original draft, Methodology. TZ: Writing – review & editing, Writing – original draft, Software, Formal analysis. WD: Writing – review & editing, Writing – original draft, Software, Formal analysis. CT: Writing – review & editing, Writing – original draft, Methodology, Formal analysis. KX: Writing – review & editing, Writing – original draft, Methodology. JM: Writing – review & editing, Writing – original draft, Validation, Formal analysis. WH: Writing – review & editing, Writing – original draft, Conceptualization. JW: Writing – review & editing, Writing – original draft, Conceptualization. SC: Writing – review & editing, Writing – original draft, Conceptualization.

## References

[ref1] Arroll B. Wallace H. B. Mount V. Humm S. P. Kingsford D. W. (2017). A systematic review and meta-analysis of treatments for acrophobia. Med. J. Aust. 206, 263–267. doi: 10.5694/mja16.00540, PMID: 28359010

[ref2] Baker A. H. Siegel L. S. (2010). Emotional freedom techniques (EFT) reduces intense fears: a partial replication and extension of Wells, Polglase, Andrews, Carrington, & Baker (2003). Energy Psychol. Theory Res. Treat. 2, 15–32. doi: 10.9769/EPJ.2010.2.2.AHB.LSS

[ref3] Borkar C. D. Fadok J. P. (2021). A novel Pavlovian fear conditioning paradigm to study freezing and flight behavior. J. Vis. Exp. 5:167. doi: 10.3791/61536, PMID: 33491674 PMC8593929

[ref4] Brandt T. Kugler G. Schniepp R. Wuehr M. Huppert D. (2015). Acrophobia impairs visual exploration and balance during standing and walking. Ann. N. Y. Acad. Sci. 1343, 37–48. doi: 10.1111/nyas.12692, PMID: 25722015

[ref5] Burger A. M. Verkuil B. Van Diest I. Van der Does W. Thayer J. F. Brosschot J. F. (2016). The effects of transcutaneous vagus nerve stimulation on conditioned fear extinction in humans. Neurobiol. Learn. Mem. 132, 49–56. doi: 10.1016/j.nlm.2016.05.007, PMID: 27222436

[ref6] Chatwin H. Stapleton P. Porter B. Devine S. Sheldon T. (2016). The effectiveness of cognitive behavioral therapy and emotional freedom techniques in reducing depression and anxiety among adults: a pilot study. Integr. Med. 15, 27–34. PMID: 27330487 PMC4898279

[ref7] Christianson J. P. (2021). The head and the heart of fear. Science 374, 937–938. doi: 10.1126/science.abm6790, PMID: 34793218

[ref8] Church D. (2010). The effect of EFT (emotional freedom techniques) on athletic performance: a randomized controlled blind trial. Open Sports Sci. J. 2, 94–99. doi: 10.2174/1875399X00902010094

[ref9] Church D. Stapleton P. Vasudevan A. O'Keefe T. (2022). Clinical EFT as an evidence-based practice for the treatment of psychological and physiological conditions: a systematic review. Front. Psychol. 13:951451. doi: 10.3389/fpsyg.2022.951451, PMID: 36438382 PMC9692186

[ref10] Clarke P. J. F. Van Bockstaele B. Marinovic W. Howell J. A. Boyes M. E. Notebaert L. (2020). The effects of left DLPFC tDCS on emotion regulation, biased attention, and emotional reactivity to negative content. Cogn. Affect. Behav. Neurosci. 20, 1323–1335. doi: 10.3758/s13415-020-00840-2, PMID: 33123862

[ref11] Clond M. (2016). Emotional freedom techniques for anxiety. J. Nerv. Ment. Dis. 204, 388–395. doi: 10.1097/NMD.0000000000000483 26894319

[ref12] Dincer B. Inangil D. (2021). The effect of emotional freedom techniques on nurses' stress, anxiety, and burnout levels during the COVID-19 pandemic: a randomized controlled trial. Explore 17, 109–114. doi: 10.1016/j.explore.2020.11.012, PMID: 33293201 PMC7834511

[ref13] Etkin A. Wager T. D. (2007). Functional neuroimaging of anxiety: a Meta-analysis of emotional processing in PTSD, social anxiety disorder, and specific phobia. Am. J. Psychiatry 164, 1476–1488. doi: 10.1176/appi.ajp.2007.07030504, PMID: 17898336 PMC3318959

[ref14] Feinstein D. (2022). Uses of energy psychology following catastrophic events. Front. Psychol. 13:856209. doi: 10.3389/fpsyg.2022.856209, PMID: 35548526 PMC9084314

[ref15] Fu Q. Liu M. Zhang L. Yang H. Zhang L. Yang S.. (2022). Head acupuncture plus Schuell’s language rehabilitation for post-stroke aphasia: a systematic review and meta-analysis of 32 randomized controlled trials. Chin. J. Integr. Med. 28, 743–752. doi: 10.1007/s11655-022-3722-5, PMID: 35907173

[ref16] Geisler F. C. M. Kubiak T. Siewert K. Weber H. (2013). Cardiac vagal tone is associated with social engagement and self-regulation. Biol. Psychol. 93, 279–286. doi: 10.1016/j.biopsycho.2013.02.013, PMID: 23466587

[ref17] Gross J. J. (2015). Emotion regulation: current status and future prospects. Psychol. Inq. 26, 1–26. doi: 10.1080/1047840X.2014.940781

[ref18] Hodgson T. (2019). "The relationship between spatial attention and eye movements," Switzerland: Springer International Publishing AG, 255–278. 10.1007/7854_2019_95 31037554

[ref19] Huppert D. Grill E. Brandt T. (2013). Down on heights? One in three has visual height intolerance. J. Neurol. 260, 597–604. doi: 10.1007/s00415-012-6685-1, PMID: 23070463

[ref20] Jasubhai D. S. Mukundan P. C. R. (2018). Cognitive Behavioural therapy and emotional freedom technique in reducing anxiety and depression in Indian adults. Int. J. Emerg. Ment. Health 20:403. doi: 10.4172/1522-4821.1000403

[ref21] Kaczkurkin A. N. Foa E. B. (2015). Cognitive-behavioral therapy for anxiety disorders: an update on the empirical evidence. Dialogues Clin. Neurosci. 17, 337–346. doi: 10.31887/DCNS.2015.17.3/akaczkurkin, PMID: 26487814 PMC4610618

[ref22] Kappelmann N. Suesse M. Steudte-Schmiedgen S. Kaldewaij R. Browning M. Michael T.. (2020). D-cycloserine as adjunct to brief computerised CBT for spider fear: effects on fear, behaviour, and cognitive biases. J. Behav. Ther. Exp. Psychiatry 68:101546. doi: 10.1016/j.jbtep.2019.101546, PMID: 31951819

[ref23] Kok B. E. Coffey K. A. Cohn M. A. Catalino L. I. Vacharkulksemsuk T. Algoe S. B.. (2013). How positive emotions build physical health. Psychol. Sci. 24, 1123–1132. doi: 10.1177/0956797612470827 23649562

[ref24] Laborde S. Mosley E. Thayer J. F. (2017). Heart rate variability and cardiac vagal tone in psychophysiological research – recommendations for experiment planning, data analysis, and data reporting. Front. Psychol. 8:213. doi: 10.3389/fpsyg.2017.00213, PMID: 28265249 PMC5316555

[ref25] Lane J. (2009). The neurochemistry of counterconditioning: acupressure desensitization in psychotherapy. Energy Psychol. 1, 31–44.

[ref26] Larsson C. E. Cabassut V. Peretout P. Marliere S. Vautrin E. Piliero N.. (2023). Assessment of the objective effect of virtual reality for preoperative anxiety in interventional cardiology. Am. J. Cardiol. 205, 207–213. doi: 10.1016/j.amjcard.2023.07.130, PMID: 37611412

[ref27] LeBeau R. T. Glenn D. Liao B. Wittchen H. Beesdo-Baum K. Ollendick T.. (2010). Specific phobia: a review of DSM-IV specific phobia and preliminary recommendations for DSM-V. Depress. Anxiety 27, 148–167. doi: 10.1002/da.20655, PMID: 20099272

[ref28] Lipponen J. A. Tarvainen M. P. (2019). A robust algorithm for heart rate variability time series artefact correction using novel beat classification. J. Med. Eng. Technol. 43, 173–181. doi: 10.1080/03091902.2019.1640306, PMID: 31314618

[ref29] Lissek S. Rabin S. Heller R. E. Lukenbaugh D. Geraci M. Pine D. S.. (2010). Overgeneralization of conditioned fear as a pathogenic marker of panic disorder. Am. J. Psychiatry 167, 47–55. doi: 10.1176/appi.ajp.2009.09030410, PMID: 19917595 PMC2806514

[ref30] Lissek S. Rabin S. J. McDowell D. J. Dvir S. Bradford D. E. Geraci M.. (2009). Impaired discriminative fear-conditioning resulting from elevated fear responding to learned safety cues among individuals with panic disorder. Behav. Res. Ther. 47, 111–118. doi: 10.1016/j.brat.2008.10.017, PMID: 19027893 PMC2758527

[ref31] Liu P. Li C. Lu Y. Liu Q. (2020). A case report of acupuncture at Neiguan point (P6) for paroxysmal supraventricular tachycardia. Complement. Med. Res. 27, 364–368. doi: 10.1159/000506360, PMID: 32224620

[ref32] Liu J. Lin L. Wang D. V. (2021). Representation of fear of heights by basolateral amygdala neurons. J. Neurosci. 41, 1080–1091. doi: 10.1523/JNEUROSCI.0483-20.2020, PMID: 33436527 PMC7880278

[ref33] Manteghi F. Nasehi M. Zarrindast M. (2017). Precondition of right frontal region with anodal tDCS can restore the fear memory impairment induced by ACPA in male mice. EXCLI J. 16, 1–13. doi: 10.17179/excli2016-693, PMID: 28337114 PMC5318674

[ref34] Mccraty R. Shaffer F. (2015). Heart rate variability: new perspectives on physiological mechanisms, assessment of self-regulatory capacity, and health risk. Glob. Adv. Health Med. 4, 46–61. doi: 10.7453/gahmj.2014.073, PMID: 25694852 PMC4311559

[ref35] McKenzie S. Eichenbaum H. (2011). Consolidation and reconsolidation: two lives of memories? Neuron 71, 224–233. doi: 10.1016/j.neuron.2011.06.037, PMID: 21791282 PMC3145971

[ref36] McTeague L. M. Lang P. J. Laplante M. Cuthbert B. N. Shumen J. R. Bradley M. M. (2010). Aversive imagery in posttraumatic stress disorder: trauma recurrence, comorbidity, and physiological reactivity. Biol. Psychiatry 67, 346–356. doi: 10.1016/j.biopsych.2009.08.023, PMID: 19875104 PMC3747632

[ref37] Melillo P. Bracale M. Pecchia L. (2011). Nonlinear heart rate variability features for real-life stress detection. Case study: students under stress due to university examination. Biomed. Eng. Online 10:96. doi: 10.1186/1475-925X-10-96, PMID: 22059697 PMC3305918

[ref38] Menevşe Ş. Yayla A. (2023). Effect of emotional freedom technique applied to patients before laparoscopic cholecystectomy on surgical fear and anxiety: a randomized controlled trial. J. Perianesth. Nurs. 39, 93–100. doi: 10.1016/j.jopan.2023.07.006, PMID: 37804271

[ref39] Minewiser L. (2017). Six sessions of emotional freedom techniques remediate one Veteran's combat-related post-traumatic stress disorder. Med. Acupunct. 29, 249–253. doi: 10.1089/acu.2017.1216, PMID: 28874927 PMC5576207

[ref40] Mittelstädt V. Miller J. (2020). Beyond mean reaction times: combining distributional analyses with processing stage manipulations in the Simon task. Cogn. Psychol. 119:101275. doi: 10.1016/j.cogpsych.2020.101275, PMID: 32032900

[ref41] Nader K. Schafe G. E. Le Doux J. E. (2000). Fear memories require protein synthesis in the amygdala for reconsolidation after retrieval. Nature 406, 722–726. doi: 10.1038/35021052, PMID: 10963596

[ref42] Napadow V. Dhond R. Park K. Kim J. Makris N. Kwong K. K.. (2009). Time-variant fMRI activity in the brainstem and higher structures in response to acupuncture. Neuroimage 47, 289–301. doi: 10.1016/j.neuroimage.2009.03.060, PMID: 19345268 PMC2692758

[ref43] Ogura K. Sugano M. Takabatake S. Naitoh Y. Nakaoka K. Data from: VR application for visual field measurement of unilateral spatial neglect patients using eye tracking. In The 7th IEEE International Conference on Healthcare Informatics. Xi'an, China: IEEE. (2019)

[ref44] Pan J. Tompkins W. J. (1985). A real-time QRS detection algorithm. I.E.E.E. Trans. Biomed. Eng. 32, 230–236. doi: 10.1109/TBME.1985.325532 3997178

[ref45] Pennington Z. T. Fanselow M. S. (2018). Indirect targeting of subsuperficial brain structures with transcranial magnetic stimulation reveals a promising way forward in the treatment of fear. Biol. Psychiatry 84, 80–81. doi: 10.1016/j.biopsych.2018.05.003, PMID: 31178062

[ref46] Piskorski J. (2005). Filtering Poincare plots. Comp. Meth. Sci. Tech. 11, 39–48. doi: 10.12921/cmst.2005.11.01.39-48

[ref47] Pittig A. Wong A. H. K. Glück V. M. Boschet J. M. (2020). Avoidance and its bi-directional relationship with conditioned fear: mechanisms, moderators, and clinical implications. Behav. Res. Ther. 126:103550. doi: 10.1016/j.brat.2020.103550, PMID: 31981801

[ref48] Powers M. B. de Kleine R. A. Smits J. A. J. (2017). Core mechanisms of cognitive behavioral therapy for anxiety and depression: a review. Psychiatr. Clin. North Am. 40, 611–623. doi: 10.1016/j.psc.2017.08.010, PMID: 29080589

[ref49] Quintana D. S. Heathers J. A. J. (2014). Considerations in the assessment of heart rate variability in biobehavioral research. Front. Psychol. 5:805. doi: 10.3389/fpsyg.2014.00805, PMID: 25101047 PMC4106423

[ref50] Rachman S. (2015). The evolution of behaviour therapy and cognitive behaviour therapy. Behav. Res. Ther. 64, 1–8. doi: 10.1016/j.brat.2014.10.006 25462876

[ref51] Rahmi T. (2013). Efektivitas Emotional Freedom Technique dalam Mengatasi Trauma Gempa Ibu Rumah Tangga. Pedagogi 12, 107–114. doi: 10.24036/pedagogi.v12i2.2212

[ref52] Reinecke A. Waldenmaier L. Cooper M. J. Harmer C. J. (2013). Changes in automatic threat processing precede and predict clinical changes with exposure-based cognitive-behavior therapy for panic disorder. Biol. Psychiatry 73, 1064–1070. doi: 10.1016/j.biopsych.2013.02.005, PMID: 23510582

[ref53] Sebastian B. Nelms J. (2017). The effectiveness of emotional freedom techniques in the treatment of posttraumatic stress disorder: a Meta-analysis. Explore 13, 16–25. doi: 10.1016/j.explore.2016.10.001, PMID: 27889444

[ref54] Shaffer F. Ginsberg J. P. (2017). An overview of heart rate variability metrics and norms. Front. Public Health 5:258. doi: 10.3389/fpubh.2017.00258, PMID: 29034226 PMC5624990

[ref55] Smith C. A. Armour M. Lee M. S. Wang L. Hay P. J. (2018). Acupuncture for depression. Cochrane Database Syst. Rev. 2018:CD004046. doi: 10.1002/14651858.CD004046.pub4, PMID: 29502347 PMC6494180

[ref56] Souto D. Kerzel D. (2021). Visual selective attention and the control of tracking eye movements: a critical review. J. Neurophysiol. 125, 1552–1576. doi: 10.1152/jn.00145.2019, PMID: 33730516

[ref57] Stapleton P. Kip K. Church D. Toussaint L. Footman J. Ballantyne P.. (2023). Emotional freedom techniques for treating post traumatic stress disorder: an updated systematic review and meta-analysis. Front. Psychol. 14:1195286. doi: 10.3389/fpsyg.2023.1195286, PMID: 37637920 PMC10447981

[ref58] Szmit M. Krajewski R. Rudnicki J. Agrawal S. (2023). Application and efficacy of transcutaneous electrical acupoint stimulation (TEAS) in clinical practice: a systematic review. Adv. Clin. Exp. Med. 32, 1063–1074. doi: 10.17219/acem/159703, PMID: 37026972

[ref59] Thayer J. F. Åhs F. Fredrikson M. Sollers J. J. Wager T. D. (2012). A meta-analysis of heart rate variability and neuroimaging studies: implications for heart rate variability as a marker of stress and health. Neurosci. Biobehav. Rev. 36, 747–756. doi: 10.1016/j.neubiorev.2011.11.009, PMID: 22178086

[ref60] Thayer J. F. Hansen A. L. Saus-Rose E. Johnsen B. H. (2009). Heart rate variability, prefrontal neural function, and cognitive performance: the Neurovisceral integration perspective on self-regulation, adaptation, and health. Ann. Behav. Med. 37, 141–153. doi: 10.1007/s12160-009-9101-z, PMID: 19424767

[ref61] Thayer J. F. Lane R. D. (2009). Claude Bernard and the heart–brain connection: further elaboration of a model of neurovisceral integration. Neurosci. Biobehav. Rev. 33, 81–88. doi: 10.1016/j.neubiorev.2008.08.004, PMID: 18771686

[ref62] van der Veen S. Bordeleau M. Pidcoe P. France C. Thomas J. (2019). Agreement analysis between Vive and Vicon systems to monitor lumbar postural changes. Sensors 19:3632. doi: 10.3390/s19173632, PMID: 31438520 PMC6749183

[ref63] Wang H. Wright B. Tindall L. Cooper C. Biggs K. Lee E.. (2022). Cost and effectiveness of one session treatment (OST) for children and young people with specific phobias compared to multi-session cognitive behavioural therapy (CBT): results from a randomised controlled trial. BMC Psychiatry 22:547. doi: 10.1186/s12888-022-04192-8, PMID: 35962334 PMC9372970

[ref64] Watson D. Clark L. A. Tellegen A. (1988). Development and validation of brief measures of positive and negative affect: the PANAS scales. J. Pers. Soc. Psychol. 54, 1063–1070. doi: 10.1037//0022-3514.54.6.1063, PMID: 3397865

[ref65] Wittfoth D. Beise J. Manuel J. Bohne M. Wittfoth M. (2022). Bifocal emotion regulation through acupoint tapping in fear of flying. Neuroimage Clin. 34:102996. doi: 10.1016/j.nicl.2022.102996, PMID: 35378497 PMC8980501

[ref66] Wittfoth D. Pfeiffer A. Bohne M. Lanfermann H. Wittfoth M. (2020). Emotion regulation through bifocal processing of fear inducing and disgust inducing stimuli. BMC Neurosci. 21:47. doi: 10.1186/s12868-020-00597-x, PMID: 33225884 PMC7681990

[ref67] Wolitzky-Taylor K. B. Horowitz J. D. Powers M. B. Telch M. J. (2008). Psychological approaches in the treatment of specific phobias: a meta-analysis. Clin. Psychol. Rev. 28, 1021–1037. doi: 10.1016/j.cpr.2008.02.007, PMID: 18410984

[ref68] Wout M. Longo S. M. Reddy M. K. Philip N. S. Bowker M. T. Greenberg B. D. (2017). Transcranial direct current stimulation may modulate extinction memory in posttraumatic stress disorder. Brain Behav. 7:e00681. doi: 10.1002/brb3.681, PMID: 28523223 PMC5434186

[ref69] Wroblewski D. Francis B. A. Sadun A. Vakili G. Chopra V. (2014). Testing of visual field with virtual reality goggles in manual and visual grasp modes. Biomed. Res. Int. 2014, 1–10. doi: 10.1155/2014/206082, PMID: 25050326 PMC4090491

[ref70] Yang J. Tang M. Cong L. Sun J. Guo D. Zhang T.. (2023). Development and validation of an assessment index for quantifying cognitive task load in pilots under simulated flight conditions using heart rate variability and principal component analysis. Ergonomics 6, 1–11. doi: 10.1080/00140139.2023.2229075, PMID: 37365918

[ref71] Ye Z. Zhu L. Li X. Gao H. Wang J. Wu S.. (2023). PC6 electroacupuncture reduces stress-induced autonomic and neuroendocrine responses in rats. Heliyon 9:e15291. doi: 10.1016/j.heliyon.2023.e15291, PMID: 37095918 PMC10121450

[ref72] Zaccaro A. Piarulli A. Laurino M. Garbella E. Menicucci D. Neri B.. (2018). How breath-control can change your life: a systematic review on psycho-physiological correlates of slow breathing. Front. Hum. Neurosci. 12:353. doi: 10.3389/fnhum.2018.00353, PMID: 30245619 PMC6137615

[ref73] Zhou W. Deng Q. Jia L. Zhao H. Yang M. Dou G.. (2020). Acute effect of transcutaneous Electroacupuncture on Globus Pharyngeus: a randomized, single-blind, crossover trial. Front. Med. 7:179. doi: 10.3389/fmed.2020.00179, PMID: 32528966 PMC7247858

